# Stigma and psychological health in psoriasis patients based on the dual-factor model of mental health: the chain mediating roles of social appearance anxiety and alexithymia

**DOI:** 10.3389/fpsyt.2024.1499714

**Published:** 2024-12-24

**Authors:** Lijun Huang, Ziyou Feng, Chengfeng Xu, Yuan Liao, Yu Yan, Chenfan Yang, Yu Li, Chun Li

**Affiliations:** ^1^ School of Nursing, Guangzhou University of Chinese Medicine, Guangzhou, China; ^2^ Department of Orthopedics, Beijing University of Chinese Medicine Shenzhen Hospital(Longgang), Shenzhen, China

**Keywords:** psoriasis, stigma, social appearance anxiety, alexithymia, psychological health

## Abstract

**Background:**

Patients with psoriasis also often experience stigma due to skin lesions, and this stigma further leads to severe psychological problems such as anxiety and depression. However, it is unclear how, and under what conditions, stigma relates to mental health. This study aimed to investigate the current status and interrelationships between stigma, social appearance anxiety, alexithymia, and mental health in patients with psoriasis. It also sought to identify the factors that influenced their mental health and to examine the mediating roles of social appearance anxiety and alexithymia in the relationship between stigma and psychological health.

**Method:**

From June to December 2023, patients with psoriasis were recruited from the outpatient department or ward of the dermatology department of a tertiary hospital in Guangzhou. Patients were assessed using the General Information Questionnaire, the Psoriasis Stigma Scale, the Social Appearance Anxiety Scale, the Toronto Alexithymia Scale, and the Satisfaction with Life Scale. Structural equation modeling (SEM) was conducted using Amos 24.0 to explore the relationships among the variables, and mediation effects were tested using SPSS 26.0.

**Results:**

A total of 317 psoriasis patients were recruited to participate in the survey. The total score of stigma of patients was (82.03 ± 1.52), which was at a moderate level. The total score of social appearance anxiety scale was (49.38 ± 1.00), which was at a high level. The total score of negative mental health of patients was (2.77 ± 0.14), which was at a low level. The total score of positive mental health of patients was (20.14 ± 0.36), which was at a medium level. The findings revealed that social appearance anxiety and alexithymia play significant chain mediating roles between stigma and negative mental health in patients with psoriasis, with an effect size of -0.031. Similarly, these factors also mediate the relationship between stigma and positive mental health, with an effect size of 0.056.

**Conclusion:**

Stigma in patients with psoriasis can directly impact their mental health and can also influence it indirectly through social appearance anxiety and alexithymia. Both social appearance anxiety and alexithymia serve as mediators in the relationship between stigma and mental health in these patients.

## 1 Introduction

Psoriasis is a common chronic inflammatory skin disorder characterized by erythematous plaques covered with silvery scales and well-demarcated borders (1). The global prevalence ranges from 0.14% to 1.99% (2), while in China, it ranges from 0.27% to 0.47% (3, 4). Psoriasis is a persistent dermatological condition that is difficult to cure and prone to relapse. It is characterized by red scaly plaques appearing on most of the skin of the body (5), and even manifests as desquamation, pustular rash, etc. (6), affecting the appearance of patients. Besides the physiological discomfort such as pruritus, pain and skin lesions (7), the majority of patients perceive others’ stereotypical impressions of them as “contagious” (8) and 27.9% experienced stigma (9),ultimately leading to psychological burden. This psychological burden can lead to severe anxiety and depression (10). The risk of comorbid mental disorders in patients with psoriasis is higher than that in the general population (11). The main reported mental disorders include schizophrenia, bipolar disorder, depression, anxiety disorder and personality disorder (12). Among patients with psoriasis, the prevalence of anxiety and depression can range from 11.52% to 27.00% (13–15). These disorders can affect people’s ability to work, reduce their quality of life, and ultimately lead to short-term disability (13). Studies have indicated that psychological factors play a crucial role in the onset and exacerbation of psoriasis, with stigma being one of the most significant factors (16). Mental health experts widely agree that many psoriasis patients struggle to adhere to treatment due to the inescapable stigma, which may even lead to a higher risk of suicide (17). Therefore, effectively alleviating the stigma associated with psoriasis is essential in the treatment process.

Due to visible disfigurement and skin lesions, psoriasis patients often feel embarrassment and fear judgment from others about their appearance, leading to social anxiety and persistent social avoidance (18). This anxiety related to physical appearance and fear of negative evaluation by others is known as Social Appearance Anxiety (19). Studies have found that patients with psoriasis have a higher degree of social appearance anxiety than ordinary people, which can eventually lead to psychological distress and affect mental health (18).In dealing with psychological distress and negative emotions, the inability to communicate and express effectively with family members gradually leads to difficulties in recognizing one’s own emotions and clearly describing and expressing one’s feelings, resulting in alexithymia, which further exacerbates the psychological disorders of patients (20, 21). Alexithymia is considered a personality trait characterized by a decreased ability to identify and verbally express emotions, a limited imaginative capacity, and externally oriented thinking (22). Alexithymia can weaken patients’ ability to manage chronic diseases. When facing chronic diseases such as respiratory diseases, patients often encounter emotional distress. The presence of alexithymia may increase the difficulty of coping with these emotional challenges. Ultimately, alexithymia may lead to a decrease in patients’ compliance with managing chronic diseases, impacting their quality of life, and potentially increasing the risk of mental health issues such as depression and anxiety, affecting their psychological well-being (23). In the field of skin diseases, alexithymia is also related to psoriasis. Patients with psoriasis often lack effective emotional expression and support in the face of psychosocial stress and appearance problems, resulting in a higher prevalence of alexithymia (24), which also directly leads to the aggravation of the psychological burden of patients and affects their mental health (25). Severe psychological problems (such as anxiety and depression) may lead to the production of inflammatory cytokines in the body, increased levels of corticotropin-releasing hormone, adrenocorticotropic hormone, and cortisol, affecting skin changes and aggravating psoriasis (26). Hence, psychological factors are a significant trigger in the onset of psoriasis.

Historically, psychological health assessments have predominantly focused on psychopathology, taking the symptoms of mental illness as the diagnostic criteria (such as anxiety and depression), and the judgment dimension was too single (27), often overlooking individuals’ inherent potential and strengths, resulting in a somewhat incomplete evaluation. Driven by the background of positive psychology, the dual-factor model of mental health came into being. Subjective Well-Being and Psychopathology are simultaneously incorporated into a model (28).The dual-factor model of mental health offers a more comprehensive assessment framework by integrating positive psychological indicators with negative ones. This approach departs from the traditional reliance solely on psychopathological symptoms, emphasizing that mental health encompasses not only the absence of psychopathological symptoms but also the holistic well-being achieved through the realization of one’s potential and strengths. By employing this model, it becomes possible to differentiate individuals who require well-being enhancement to prevent psychological disorders from those who need professional intervention, based on the dimensions of psychopathology and subjective well-being.

Given the aforementioned considerations, this study aimed to elucidate the levels of stigma, social appearance anxiety, alexithymia, and psychological well-being among psoriasis patients. By analyzing the impact of stigma, social appearance anxiety, and alexithymia on psychological well-being, and exploring the potential pathways through which these factors interrelate, this research provides crucial insights. The findings will aid families, healthcare providers, and society in identifying individuals at heightened risk for psychological health issues. Furthermore, this study will offer a valuable reference for healthcare professionals in developing tailored psychological interventions, ultimately enhancing the psychological health and quality of life of psoriasis patients.

## 2 Materials and methods

### 2.1 Participants

A convenience sampling method was employed to select psoriasis patients from the dermatology outpatient clinic and inpatient department of a tertiary hospital in Guangzhou between June and December 2023. The inclusion criteria were: a confirmed diagnosis of psoriasis; age ≥18 years; mentally alert and able to communicate effectively; capable of completing the questionnaire independently or with assistance from the researcher; and providing informed consent and willingness to participate in the study. The exclusion criteria included: the presence of other severe chronic diseases (e.g. heart, lung, or renal failure); cognitive impairment or other psychiatric disorders that would prevent completion of the survey.

### 2.2 Procedures

First, the researchers participating in the study received a uniform training. Before patients with psoriasis fill out the questionnaire, the investigators will explain the purpose, method, and significance of the study. Then, the patients themselves were asked if they would like to participate in the study and informed that completing the questionnaire was anonymous. With permission from the patient, they officially started filling after they signed a written informed consent. All patients filled out the questionnaire alone and retrieved it on site, which took approximately 20 minutes to complete. If the questionnaire was found incomplete or excessive repeated selection, it will not be used. We invited 330 patients to participate in the study, of which 7 patients did not complete the questionnaire and 6 patients had too many repeated options on the questionnaire and were judged to be invalid as above. Consequently, 317 valid questionnaires were collected, yielding an effective response rate of 96.6%.

### 2.3 Measure

#### 2.3.1 General information questionnaire

Based on a comprehensive literature review, 14 demographic and clinical variables were selected for inclusion in the study of psoriasis patients. These variables are: age, occupation, gender, educational level, marital status, income, place of residence, type of health insurance, type of psoriasis, location of skin lesions, duration of disease, severity of skin lesions, presence of pruritus, and family history of psoriasis.

#### 2.3.2 Psoriasis patients’ stigma experience questionnaire

The Feelings of Stigmatization Questionnaire (FSQ) is a self-assessment tool developed by Ginsburg IH et al. (29) based on the conceptual model of stigma and the perceived experiences of patients with psoriasis. It is designed to evaluate the stigma experienced by individuals with psoriasis. The scale consists of 6 dimensions: anticipation of rejection, feelings of being flawed, sensitivity to the opinions of others, guilt and shame, positive attitudes, and secretiveness. Chinese scholars, including Yan X et al. (30), have translated and adapted the FSQ for use in China. The Chinese version of FSQ consists of four dimensions: sensitivity and imperfections, foreboding of rejection, secretiveness, and positivity. Its Cronbach’s alpha coefficient ranges from 0.73 to 0.90, indicating good reliability. It is currently widely utilized among psoriasis patients in China (31). The scale was scored using a 6-point Likert scale ranging from 1 (strongly agree) to 6 (strongly disagree), with lower scores indicating higher levels of stigma in patients. In our study, the Cronbach’s α for the total score of FSQ was 0.937, and the Cronbach’s α for each dimension was 0.937, 0.888, 0.792, 0.702, respectively.

#### 2.3.3 Social appearance anxiety scale

The Social Appearance Anxiety Scale (SAAS) was developed by Hart TA et al. (19) in 2008 to measure the level of social appearance anxiety in individuals. It was subsequently translated and culturally adapted for use in China by Kong S et al. (32) in 2009. The SAAS is a unidimensional scale consisting of 16 items, each rated on a 5-point Likert scale from 1 (not at all) to 5 (extremely). The Chinese version of the scale has demonstrated high reliability, with a Cronbach’s alpha coefficient of 0.911. The total score ranges from 16 to 80, with higher scores indicating greater levels of social appearance anxiety. In our study, the Cronbach’s α for SAAS was 0.961.

#### 2.3.4 Toronto alexithymia scale

The Toronto Alexithymia Scale (TAS-20) was initially developed by Taylor GJ (33) in 1984 and subsequently revised by Bagby RM et al. (34) in 1994. The TAS-20 encompasses three dimensions: difficulty identifying feelings, difficulty describing feelings, and externally oriented thinking. The TAS-20 uses a 5-point Likert scale ranging from 1 (strongly disagree) to 5 (strongly agree), with higher scores representing greater alexithymia. Subsequently, Yuan YG et al. (35) translated the TAS-20 Chinese version in 2004.The scale demonstrates good reliability, with an overall Cronbach’s alpha coefficient of the Chinese version of 0.83. In our study, the Cronbach’s α for the total score of TAS-20 was 0.909, and the Cronbach’s α for each dimension was 0.904, 0.727, 0.712, respectively.

#### 2.3.5 General health questionnaire

The General Health Questionnaire (GHQ-12) was originally developed by Goldberg DP et al. (36) in the 1970s. The version employed in this study was adapted by Zheng TA (37), a scholar from Taiwan, China, incorporating cultural characteristics specific to China. The Cronbach’s α of the Chinese version of GHQ-12 was 0.83. The GHQ-12 comprises three dimensions: somatic symptoms, anxiety and worry, and depression. This questionnaire reflects the psychological health status of the patient based on self-assessment, consisting of 12 questions. Each question has four options: A, B, C, and D. The scoring method is a bimodal system (0-0-1-1), where selecting A or B is assigned 0 points, and selecting C or D is assigned 1 point. The higher the score, the higher the likelihood of developing psychological disorders. The Cronbach’s α was 0.798 in our study.

#### 2.3.6 Life satisfaction scale

The Life Satisfaction Scale, developed by Diener E et al. (38), is an effective tool for measuring individuals’ life satisfaction. It was subsequently translated and culturally adapted for use in China by Xiong C et al. (39).The scale is a unidimensional scale consisting of 5 items, each rated on a 7-point Likert scale from 1 (strongly disagree) to 7 (strongly agree).The level of life satisfaction is determined by the scale’s scores, with higher scores indicating greater life satisfaction. The Cronbach’s α coefficient of the Chinese version of the scale was 0.78, and the Cronbach’s α coefficient of this study was 0.918.

### 2.4 Data analysis

Confirmatory factor analysis was performed using Amos 24.0. Data were processed using SPSS 26.0. For normally distributed metric data, the mean ± standard deviation was used, whereas the median and interquartile range were employed for non-normally distributed data. Categorical data were described using frequencies and percentages. Independent samples t-tests or one-way ANOVA were used for normally distributed continuous variables, while the rank-sum test was applied for non-normally distributed data. Differences in demographic characteristics among psoriasis patients were analyzed. Pearson correlation analysis was used for normally distributed data, and Spearman rank correlation was employed for non-normally distributed data. The PROCESS 3.5 macro in SPSS 26.0 was utilized to test for mediation effects, specifically analyzing whether social appearance anxiety and alexithymia mediate the relationship between stigma and psychological health in psoriasis patients.

### 2.5 Ethical principles

This study has obtained ethical approval from the hospital ethics committee (YE2023-230-01). In strict adherence to the principles of informed consent and voluntary participation, the purpose, significance, and voluntary nature of the investigation were disclosed to the participants. Following the participants’ informed consent, the questionnaire survey was conducted, and participants were free to choose to leave and not participate in the survey without any repercussions. The study was conducted anonymously, with no information collected that could identify the participants. The data collected were used solely for this research and subjected to strict confidentiality measures.

## 3 Results

### 3.1 Common method bias

To assess the potential artificial covariance between predictor and criterion variables caused by the same data source, measurement environment, item context, and item characteristics, this study utilized Harman’s single-factor test to evaluate common method bias. An unrotated exploratory factor analysis was performed using SPSS 26.0 on all items from the Psoriasis Stigma Experience Questionnaire, Social Appearance Anxiety Scale, Toronto Alexithymia Scale, Chinese Health Questionnaire, and Life Satisfaction Scale. The analysis revealed 13 factors with eigenvalues greater than 1, with the first factor accounting for 30.46% of the variance, which is below the critical threshold of 50% (40). Therefore, significant common method bias is not present in this study.

### 3.2 Baseline data

The equations should be inserted in editable format from the equation editor. Among the 317 psoriasis patients included in the analysis, 172 were male (54.26%) and 145 were female (45.74%), with an age range of 18 to 76 years and a mean age of 39.74 ± 13.61 years. The majority of patients were aged 18 to 45 years (68.13%). The educational level of psoriasis patients was generally high, with 47.00% having a bachelor’s degree or higher. Most patients resided in urban areas (82.00%). The predominant type of psoriasis was the vulgaris form (92.74%), and 48.26% of patients had lesions covering their entire body. The duration of the disease was less than 10 years for most patients (45.43%). The severity of psoriasis was mostly mild (46.69%), with a high prevalence of itching (76.03%). Additionally, the majority of patients had no family history of psoriasis (79.18%). Frequency and percentage were used to statistically describe the general characteristics of psoriasis patients in this study. Details are provided in **Supplementary Table 1**.

### 3.3 Stigma, social appearance anxiety, alexithymia, and psychological health levels in psoriasis patients

In this survey of 317 psoriasis patients, the total stigma score was 82.03 ± 1.52, indicating a moderate level compared to the median score of 81 on the scale. The dimension scores, ranked from highest to lowest, were: prosocial silence (2.62 ± 0.90), negative silence (2.60 ± 0.88), defensive silence (2.52 ± 0.93), and indifferent silence (2.10 ± 0.81). The total score for social appearance anxiety was 49.38 ± 1.00, which is relatively high compared to the median score of 32 on the scale. The total score for alexithymia was 59.41 ± 0.70, also relatively high compared to the median score of 40 on the scale. The dimension scores, ranked from highest to lowest, were: externally oriented thinking (24.01 ± 0.26), difficulty identifying feelings (20.14 ± 0.32), and difficulty describing feelings (15.27 ± 0.20). The total negative psychological health score was 2.77 ± 0.14, indicating a relatively low level compared to the cutoff score of 4 for detecting psychological disorders. The total positive psychological health score was 20.14 ± 0.36, indicating a moderate level compared to the median score of 15 on the scale.

### 3.4 Analysis of psychological health in psoriasis patients

A univariate analysis was conducted with the general characteristics of psoriasis patients as independent variables and the total scores of psychological health (both negative and positive) as dependent variables. For binary variables that are normally distributed and have homogeneity of variance, t-tests were used. For multicategorical variables that are normally distributed and have homogeneity of variance, one-way ANOVA was used. For variables that are not normally distributed or do not have homogeneity of variance, the rank-sum test was employed.

The results of the univariate analysis of psychological health (both negative and positive) showed that there were statistically significant differences in psychological health among psoriasis patients in terms of age, disease duration, and disease severity. Detailed results are presented in **Supplementary Tables 2** and **3**.

### 3.5 Correlation analysis of stigma, social appearance anxiety, alexithymia, and psychological health

The correlations among the study variables were examined using Pearson correlation analysis. As shown in **Table 1**, there were significant correlations between psychological health (both positive and negative), alexithymia, psoriasis-related stigma, and social appearance anxiety (P < 0.05 or P < 0.001).

**Table 1:** Correlation analysis among study variables.

Variables	Psychological Health (negative)	Psychological Health (positive)	Alexithymia	Stigma	Social Appearance Anxiety
Psychological Health (negative)	1				
Psychological Health (positive)	-0.577^**^	1			
Alexithymia	0.538^**^	-0.444^**^	1		
Stigma	-0.432^**^	0.379^**^	-0.431^**^	1	
Social Appearance Anxiety	0.496^**^	-0.373^**^	0.441^**^	-0.648^**^	1

**Correlation is significant at the 0.01 level (2-tailed).

### 3.6 Impact of stigma on psychological health in psoriasis patients - chain mediation effect test

This study utilized the PROCESS 3.5 macro for SPSS 24.0, developed by Hayes, to conduct the statistical analysis. Model 6 was selected to test the chain mediation effect. A bootstrap sampling method with 5000 iterations and a 95% confidence interval was used to examine the chain mediation effect of social appearance anxiety and alexithymia on the relationship between psoriasis-related stigma and negative psychological health. As shown in **Table 2**, psoriasis-related stigma significantly negatively predicted social appearance anxiety (β=-0.648, t=-15.086, P<0.001) and alexithymia (β=-0.214, t=-3.269, P<0.05). Furthermore, social appearance anxiety significantly positively predicted alexithymia (β=0.303, t=4.636, P<0.001), social appearance anxiety significantly positively predicted negative psychological health (β=0.274, t=4.522, P<0.001), and alexithymia significantly positively predicted negative psychological health (β=0.383, t=7.576, P<0.001).

**Table 2:** Mediation effect test.

	Social appearance anxiety		Alexithymia		General health	
	β	*t*	β	*t*	β	*t*
Stigma	-0.648	-15.086**	-0.214	-3.269*	-0.082	-1.371
Social appearance anxiety			0.303	4.636**	0.274	4.522**
Alexithymia					0.383	7.576**
R^2^	0.419		0.221		0.376	
F	227.583		44.611		62.860	

**Correlation is significant at the 0.01 level (2-tailed); *Correlation is significant at the 0.05 level (2-tailed).

**Figure 1:**
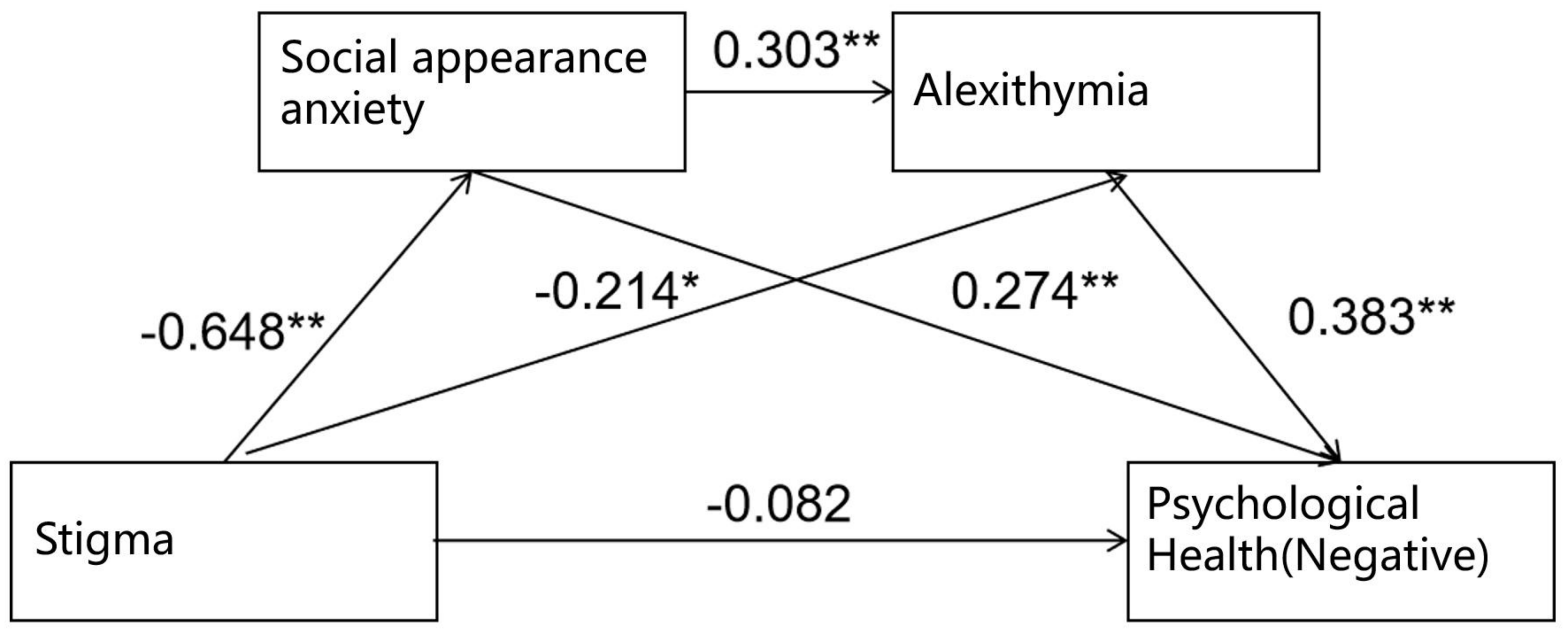
Chain mediation effect model of social appearance anxiety and alexithymia (negative). **Correlation is significant at the 0.01 level (2-tailed); *Correlation is significant at the 0.05 level (2-tailed).

A longitudinal chain mediation model was constructed with stigma as the independent variable, social appearance anxiety and alexithymia as mediating variables, and negative psychological health as the dependent variable. The chain mediation model is illustrated in **Figure 1**.

The results of the bootstrap test for the mediation effect indicated that the 95% confidence interval did not include zero, suggesting that the mediation effect was significant. The model fit results and path coefficients for the mediation effect model are presented in **Table 3**.

**Table 3:** Mediation effects and confidence intervals.

Pathway	Effect	Proportion of total effect	95% Confidence intervals	
			lower-bound	upper-bound
Stigma→social appearance anxiety→psychological health(negative)	-0.017	42.71%	-0.026	-0.009
Stigma→alexithymia→psychological health(negative)	-0.008	19.69%	-0.015	-0.002
Stigma→social appearance anxiety→alexithymia→psychological health(negative)	-0.007	18.16%	-0.012	-0.003
Total indirect effects	-0.031		-0.041	-0.023

The chain mediation effect of social appearance anxiety and alexithymia on the relationship between psoriasis-related stigma and positive psychological health was tested. As shown in **Table 4**, psoriasis-related stigma significantly negatively predicted social appearance anxiety (β=-0.648, t=-15.086, P<0.001) and alexithymia (β=-0.214, t=-3.269, P<0.05). Additionally, social appearance anxiety significantly positively predicted alexithymia (β=0.303, t=4.636, P<0.001), social appearance anxiety significantly negatively predicted positive psychological health (β=-0.150, t=-2.252, P<0.05), and alexithymia significantly negatively predicted positive psychological health (β=-0.328, t=-5.890, P<0.001).

**Table 4:** Mediation effect test.

	Social appearance anxiety		Alexithymia		Life satisfaction	
	β	*t*	β	*t*	β	*t*
Stigma	-0.648	-15.086**	-0.214	-3.269*	0.121	1.849
Social appearance anxiety			0.303	4.636**	-0.150	-2.252*
Alexithymia					-0.328	-5.890**
R^2^	0.419		0.221		0.244	
F	227.583		44.611		33.745	

**Correlation is significant at the 0.01 level (2-tailed); *Correlation is significant at the 0.05 level (2-tailed).

**Figure 2:**
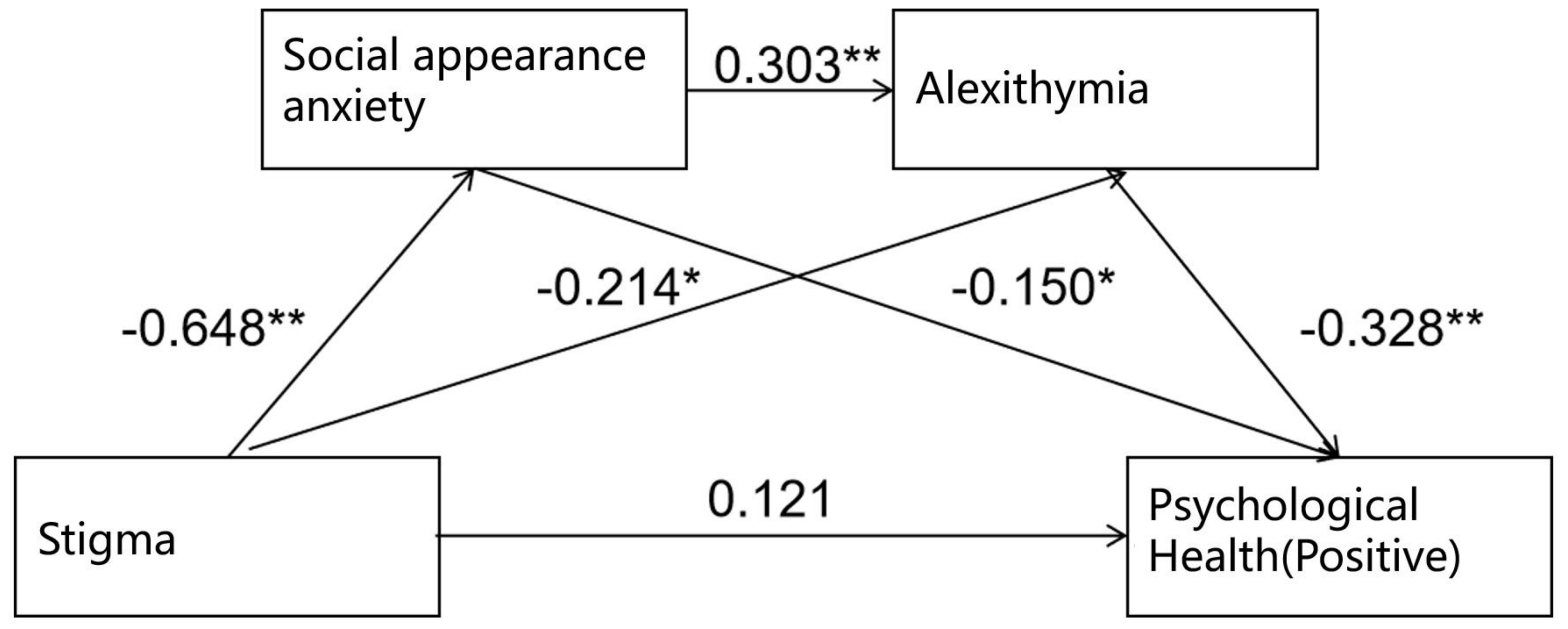
Chain mediation effect model of social appearance anxiety and alexithymia (positive). **Correlation is significant at the 0.01 level (2-tailed); *Correlation is significant at the 0.05 level (2-tailed).

A longitudinal chain mediation model was constructed with stigma as the independent variable, social appearance anxiety and alexithymia as mediating variables, and positive psychological health as the dependent variable. The chain mediation model is illustrated in **Figure 2**.

The results of the bootstrap test for the mediation effect showed that the 95% confidence interval did not include zero, indicating that the mediation effect was significant. The model fit results and path coefficients for the mediation effect model are presented in **Table 5**.

**Table 5:** Mediation effects and confidence intervals.

Pathway	Effect	Proportion of total effect	95% Confidence intervals	
			lower-bound	upper-bound
Stigma→social appearance anxiety→psychological Health(positive)	0.024	27.58%	0.002	0.049
Stigma→alexithymia→psychological Health(positive)	0.017	19.84%	0.004	0.032
Stigma→social appearance anxiety→alexithymia→psychological Health(positive)	0.016	18.19%	0.007	0.027
Total indirect effects	0.056		0.033	0.082

According to the recommendations of Wen Z et al. (41), the following indices were selected to evaluate the path coefficients of the model: χ²/df, RMSEA, CFI, TLI, and SRMR. The fit indices met the following criteria: χ²/df: 1–3, RMSEA < 0.1, CFI and TLI > 0.8, and SRMR < 0.08, indicating that the path coefficients of the model are statistically significant. The fit indices of the confirmatory factor analysis model are shown in **Table 6**.

**Table 6:** Fit indices of the confirmatory factor analysis model.

	c^2^	df	c^2^/df	RMSEA	CFI	TLI	SRMR
Negative model of mental health	5368.688	2694	1.993	0.056	0.826	0.821	0.060
Positive model of mental health	4774.409	2204	2.166	0.061	0.836	0.831	0.062

## 4 Discussion

### 4.1 Current status analysis of stigma, social appearance anxiety, alexithymia, and psychological health in psoriasis patients

The study results indicate that the stigma level among psoriasis patients is moderate, consistent with the findings of Jankowia B et al. (42).Among the dimensions, sensitivity and flaw scored the highest, followed by concealment and positive dimensions, with anticipation of rejection scoring the lowest. The negative impact of these feelings may influence individuals’ self-concept as well as their psychological and behavioral responses. According to the scoring rules of this scale, lower scores correspond to higher levels of stigma, indicating that psoriasis patients feel the strongest about anticipating rejection. This anticipation can lead to difficulties in interpersonal relationships and avoidance of certain situations due to fear of rejection. Previous studies have identified rejection experiences as significant predictors of feelings of aversion and sensitivity in psoriasis patients. These experiences can diminish patients’ attitudes toward their illness, resulting in a loss of confidence, resistance to treatment, and ultimately affecting recovery (30). The underlying reasons include: ①Rejection in job applications or romantic relationships can cause significant distress, and patients may attribute this distress to their psoriasis, further lowering their self-esteem. ②Individuals often form experiential memories after being hurt. Thus, when encountering rejection, patients may feel disliked by others, exacerbating their sensitivity and psychological stress. ③It is noteworthy that even without actual experiences of insult or rejection, the fear of discrimination or rejection may lead patients to choose avoidance as a coping strategy to reduce psychological stress and pressure. Therefore, we strongly recommend that healthcare providers actively assist patients in correctly perceiving their exposed lesions and correcting misunderstandings while communicating disease information. Additionally, helping patients overcome psychological barriers to accepting their condition can foster confidence in their ability to achieve recovery.

Psoriasis patients exhibit a moderately high level of social appearance anxiety. However, this level is lower than the findings of Zhu Y et al. (43) and Wang J et al. (44), who investigated female psoriasis patients and patients with other diseases. This suggests that, compared to men, women are more concerned about their appearance and feel more pressure regarding their physical appearance. Additionally, compared to specific groups with more apparent physical damage, such as patients undergoing mastectomy or those with facial burns, psoriasis patients have lower social appearance anxiety. This may be related to the characteristics of psoriasis itself; despite its long duration, difficulty in curing, and high recurrence rate, it can be managed by covering the affected skin and actively treating the condition to control the disease. Healthcare providers need to enhance public awareness about psoriasis by disseminating knowledge about the disease, encouraging societal support for psoriasis patients, and reducing their feelings of inferiority and loneliness. Furthermore, healthcare providers can help improve patients’ positive self-recognition, making them realize that external appearance is only one aspect of self-image while also helping to maintain a positive image. Additionally, assisting patients in accurately understanding their disease, facilitating role transitions, reducing self-conflict, and enhancing their ability to adapt to the disease will not only boost their self-confidence but also improve their social skills and interpersonal interactions (45).

Psoriasis patients exhibit a moderately high level of alexithymia, consistent with the findings of Talamonti M et al (46). The reasons for this may include concerns about their external appearance, which may lead patients to worry about the impact of the disease on their social interactions and fear of discrimination, resulting in the suppression of their emotions and reluctance to express themselves. Additionally, societal prejudice against psoriasis exacerbates the patients’ sense of shame about the disease, making it difficult for them to find appropriate individuals to confide in and adequately express their inner thoughts. Among the various dimensions, externally oriented thinking scored the highest, indicating that psoriasis patients tend to focus their emotional activities on external daily life rather than internal thoughts, displaying a pragmatic thinking tendency with excessive attention to external details. They may overly pursue external demands and place greater emphasis on external standards and evaluations. Their alexithymia often stems from interpersonal relationships, social roles, and environmental atmospheres that are closely related to them, leading to a lack of attention to their internal needs and resulting in high levels of externally oriented thinking. The underlying reasons include: ①Individuals with alexithymia have difficulty recognizing their own or others’ emotions, leading to challenges in accurately conveying personal subjective feelings during communication. These individuals often focus too much on details while neglecting the whole, negatively impacting their interpersonal relationships and social adaptability. ②The skin defects associated with psoriasis increase psychological burden and lead to social rejection, making interpersonal interactions challenging (47). Consequently, these patients are less willing to engage in proactive communication. This suggests that healthcare providers should not only impart knowledge about the disease and caregiving techniques during health education for psoriasis patients but also focus on communication skills training, encouraging patient interaction and mutual support, and sharing health-promoting knowledge and experiences. In addition to traditional dermatological treatments, methods such as behavioral relaxation therapy, suggestion therapy, social support, behavioral therapy, and self-training may also benefit the condition (48).

Psoriasis patients exhibit a relatively low level of negative psychological health, and this level is even lower than the related studies of Wang Q et al. (49) and Guo XG et al (50). The General Health Questionnaire primarily measures psychopathological indicators, including depression and anxiety. However, item analysis results indicate that the highest-scoring items are ‘Have you experienced insomnia due to excessive worry?’ and ‘Do you feel distressed or anxious?’, suggesting that while most psoriasis patients experience negative emotions, these do not reach the threshold for a psychopathological diagnosis. Most of the current research focuses on negative emotions, often using assessment tools such as the Milton Anxiety Scale, the Hamilton Depression Scale, the self-rating Depression Scale (SDS) and the Self-rating Anxiety Scale (SAS) (51). However, these studies often find patients with significant symptoms that do not meet the threshold for psychopathology. In addition, some studies have explored the potential relationship between psoriasis and anxiety and depression from the perspective of bioinformatics (52), but most of these studies have focused on major depressive disorder, while in fact, most patients with psoriasis do not reach this level of depression. The underlying reasons include: ①Some patients may have somatic symptoms, such as palpitation, palpitation, chest pain, etc., which may not be fully reflected in the assessment tools. ②The coping style of patients in the face of the disease can also affect their psychological state. Some patients may adopt positive coping styles, such as seeking social support and psychological adjustment, so as to reduce the symptoms of anxiety and depression. ③Some drugs for psoriasis may cause nervous system disorders, leading to mood swings. However, such mood swings are usually transient and do not necessarily meet diagnostic criteria for anxiety or depression. Therefore, healthcare providers must exhibit deep humanistic care towards patients. This involves effective communication, conveying empathy and concern, and understanding the suffering endured by patients. Additionally, educating patients’ families is essential so they can provide necessary social support, alleviating psychological stress and maintaining the psychological health of the patients.

Psoriasis patients exhibit a moderate level of positive psychological health, which is lower than the levels reported by Zhang X et al. (53), and colleagues for individuals with other diseases and healthy populations. This indicates that psoriasis patients have lower subjective well-being. Subjective well-being is a crucial psychological indicator of social quality of life. Factors such as the severity of psoriasis, the duration of the disease, and its chronic nature can threaten patients’ quality of life. As the disease duration increases, patients’ life satisfaction may gradually decline. Psoriasis, being a chronic inflammatory skin condition characterized by recurrent episodes, currently has no cure and involves high treatment costs. Additionally, skin lesions can negatively impact marital relationships and sexual life (54). These factors collectively can significantly reduce patients’ subjective well-being. To address this, we recommend adopting intervention strategies based on the Information-Knowledge-Belief-Behavior model to reduce anxiety and depression, alleviate body image disturbances, and mitigate the impact of the disease on patients’ quality of life (55). Moreover, we should actively explore various psychological intervention approaches, such as Rational Emotive Behavior Therapy (REBT), to enhance patients’ self-esteem and subjective well-being.

### 4.2 Analysis of the chain mediation effect of social appearance anxiety and alexithymia on the relationship between stigma and psychological health in psoriasis patients

The results of this study indicate that social appearance anxiety and alexithymia have a chain mediation effect on the relationship between stigma and psychological health in psoriasis patients, accounting for 18.16% of the total effect. The study found that social appearance anxiety positively affects alexithymia. When these patients face negative emotions, they may struggle to communicate effectively and express their feelings, leading to difficulties in emotional recognition and expressing their inner thoughts clearly. Dalbudak E et al. (56) found that high levels of anxiety and physiological symptoms induced by social anxiety lead patients to avoid sensory experiences and suppress emotional expression. In this context, alexithymia may serve as a psychological defense mechanism for social anxiety patients to maintain lower anxiety levels. Chen CH (57) and Zhang H et al. (58) also regard alexithymia and social anxiety as key mediating factors and believe that there is a close relationship between them. Relevant studies have revealed that in social situations, individuals with good emotion recognition and description are more likely to express their emotions and feelings, which is conducive to establishing good interpersonal relationships and reducing social anxiety (59).However, those with high alexithymia often show indifference and alienation in social occasions, which have a negative impact on interpersonal relationships and cause social anxiety and social communication difficulties (60). In addition, studies have found that due to the lack of imagination and introspective thinking style, people with high alexithymia often suppress themselves when facing stressful events or inner conflicts, which leads to increased anxiety (61). This emotional change, in turn, has an impact on their physical and mental health that cannot be ignored.

From a neurological perspective, functional imaging studies have shown decreased activity in the prefrontal cortex and anterior cingulate cortex in patients with social anxiety, suggesting impaired emotional processing abilities (62). Related research on alexithymia also indicates abnormal activity in these regions, implying that social anxiety patients may have deficits in emotional awareness (63). The presence of significant alexithymia symptoms in highly socially anxious individuals suggests that alexithymia is a major psychological risk factor for social anxiety (64), providing a basis for early screening of social anxiety risk. Under stressful events, patients with alexithymia may experience psychological issues such as depression, somatization, and paranoia. Highly socially anxious individuals may overuse cognitive resources due to worry and fear, affecting emotional identification and control. Therefore, individuals with high social anxiety are prone to alexithymia, characterized by difficulty in clearly expressing emotions.

Encouraging psoriasis patients to actively engage in various social activities can improve their interpersonal relationships, alleviate symptoms of social anxiety and alexithymia, and promote psychological well-being. Furthermore, fostering communication between patients and healthcare providers, peers, and family members can expand their avenues for self-expression, enabling them to obtain crucial medical information and social support. This, in turn, can ameliorate alexithymia symptoms. These interventions are instrumental in enhancing patients’ psychological health, bolstering their adaptability to the disease, and improving their overall quality of life.

In conclusion, this study investigated the relationship between stigma and psychological health, validating the mediating roles of social appearance anxiety and alexithymia. Nonetheless, the study has several limitations. First, the sample was drawn from patients at a single tertiary hospital in Guangzhou, resulting in a limited sample size and scope. Second, as a cross-sectional study, it cannot establish causal relationships between the variables, limiting the generalizability of the findings. Future research should involve larger sample sizes and broader investigation scopes to enhance the representativeness and persuasiveness of the conclusions. Moreover, longitudinal studies are recommended to track changes in patients’ psychological health over time, further clarifying the causal relationships between variables and the psychological health of psoriasis patients.

## Data Availability

The original contributions presented in the study are included in the article/**Supplementary Material**. Further inquiries can be directed to the corresponding authors.

## Supplementary material

Supplementary Table 1 General information of psoriasis patients (n=317).

Supplementary Table 2 Univariate analysis results of negative psychological health in psoriasis patients.

Supplementary Table 3 Univariate analysis results of positive psychological health in psoriasis patients.
