# Supplementary material for: Stigma and psychological health in psoriasis patients based on the dual-factor model of mental health: the chain mediating roles of social appearance anxiety and alexithymia
Source: Front Psychiatry. 2024 Dec 24;15:1499714. doi: 10.3389/fpsyt.2024.1499714 (PMC11703903; doi:10.3389/fpsyt.2024.1499714)
Supplement: Supplementary Table 1 — General information of psoriasis patients (n=317). [file Table1.docx]

Supplementary Material Table 1: General Information of Psoriasis Patients (n=317)

| Variables | Subject | Number | Percent(%) |
| --- | --- | --- | --- |
| Age | 18-45 | 216 | 68.13 |
|  | 46-69 | 92 | 29.02 |
|  | >69 | 9 | 2.83 |
| Vocational status | Be on the job | 196 | 61.82 |
|  | Other | 121 | 38.17 |
| Gender | Male | 172 | 54.26 |
|  | Female | 145 | 45.74 |
| Education level | Junior high school or below | 35 | 11.04 |
|  | High school or technical secondary school | 72 | 22.71 |
|  | Junior college | 61 | 19.24 |
|  | Bachelor degree or above | 149 | 47.00 |
| Marital status | Unmarried | 104 | 32.80 |
|  | Married | 207 | 65.30 |
|  | Other | 6 | 1.89 |
| Personal monthly income (RMB/Yuan) | <1500 | 33 | 10.41 |
|  | 1500-2999 | 27 | 8.52 |
|  | 3000-4999 | 63 | 19.87 |
|  | ≥5000 | 194 | 61.20 |
| Place of residence | Rural | 57 | 17.98 |
|  | Cities or towns | 260 | 82.02 |
| Types of medical insurance | Medical insurance | 278 | 87.70 |
|  | Out-of-pocket medical care | 39 | 12.30 |
| Types of psoriasis | Psoriasisvulgaris | 294 | 92.74 |
|  | Psoriasisarfhropathica | 10 | 3.15 |
|  | Psoriasispustulosa | 3 | 0.94 |
|  | Erythrodermicpsoriasis | 10 | 3.15 |
| Site of skin lesions | Head and face | 79 | 24.92 |
|  | The four limbs | 62 | 19.56 |
|  | Trunk | 23 | 7.26 |
|  | The whole body | 153 | 48.26 |
| Duration of disease (years) | <10 | 144 | 45.43 |
|  | 10-20 | 98 | 30.91 |
|  | >20 | 75 | 23.66 |
| Severity of illness | Mild | 148 | 46.69 |
|  | Moderate | 58 | 18.30 |
|  | Serious | 111 | 35.02 |
| Whether there is itching | Yes | 241 | 76.03 |
|  | No | 76 | 23.97 |
| Whether there is a family history | Yes | 66 | 20.82 |
|  | No | 251 | 79.18 |

Supplementary Material Table 2: Univariate Analysis Results of Negative Psychological Health in Psoriasis Patients

| Variables | Subject | Total score of the general health questionnaire | *t/F* | *P* |
| --- | --- | --- | --- | --- |
| Age | 18-45 | 4.87±2.77 | 29.446 | <0.001 |
|  | 46-69 | 3.77±2.46 |  |  |
|  | >69 | 2.38±2.09 |  |  |
| Vocational status | Be on the job | 2.48±2.29 | 1.902 | 0.080 |
|  | Other | 2.73±2.57 |  |  |
| Gender | Male | 2.62±2.49 | -1.219 | 0.224 |
|  | Female | 2.96±2.49 |  |  |
| Education level | Junior high school or below | 3.09±2.79 | 0.392 | 0.759 |
|  | High school or technical secondary school | 2.82±2.72 |  |  |
|  | Junior college | 2.89±2.50 |  |  |
|  | Bachelor degree or above | 2.63±2.31 |  |  |
| Marital status | Unmarried | 2.51±2.50 | 1.132 | 0.324 |
|  | Married | 2.92±2.48 |  |  |
|  | Other | 2.16±2.93 |  |  |
| Personal monthly income (RMB/Yuan) | <1500 | 3.48±2.87 | 1.189 | 0.320 |
|  | 1500-2999 | 2.89±2.47 |  |  |
|  | 3000-4999 | 2.95±2.76 |  |  |
|  | ≥5000 | 2.58±2.32 |  |  |
| Place of residence | Rural | 2.77±2.49 | 0.055 | 0.956 |
|  | Cities or towns | 2.77±2.44 |  |  |
| Types of medical insurance | Medical insurance | 3.33±2.42 | 0.291 | 0.832 |
|  | Out-of-pocket medical care | 2.79±2.46 |  |  |
| Types of psoriasis | Psoriasisvulgaris | 2.80±2.48 | 0.689 | 0.559 |
|  | Psoriasisarfhropathica | 2.30±2.16 |  |  |
|  | Psoriasispustulosa | 1.00±1.00 |  |  |
|  | Erythrodermicpsoriasis | 3.10±3.35 |  |  |
| Site of skin lesions | Head and face | 2.52±2.24 | 0.611 | 0.608 |
|  | The four limbs | 2.79±2.67 |  |  |
|  | Trunk | 2.48±2.43 |  |  |
|  | The whole body | 2.94±2.56 |  |  |
| Duration of disease (years) | <10 | 1.29±1.11 | 174.59 | 0.000 |
|  | 10-20 | 2.91±2.69 |  |  |
|  | >20 | 5.44±1.77 |  |  |
| Severity of illness | Mild | 1.34±1.40 | 74.68 | <0.001 |
|  | Moderate | 3.31±2.52 |  |  |
|  | Serious | 4.41±2.51 |  |  |
| Whether there is itching | Yes | 2.85±2.48 | 0.936 | 0.350 |
|  | No | 2.54±2.54 |  |  |
| Whether there is a family history | Yes | 2.32±2.36 | -1.670 | 0.096 |
|  | No | 2.89±2.52 |  |  |

Supplementary Material Table 3: Univariate Analysis Results of Positive Psychological Health in Psoriasis Patients

| Variables | Subject | Total score of the Life Satisfaction Scale | *t/F* | *P* |
| --- | --- | --- | --- | --- |
| Age | 18-45 | 16.57±6.11 | 9.452 | <0.001 |
|  | 46-69 | 18.99±6.09 |  |  |
|  | >69 | 23.44±5.68 |  |  |
| Vocational status | Be on the job | 20.50±6.30 | 0.320 | 0.926 |
|  | Other | 20.01±6.53 |  |  |
| Gender | Male | 20.41±6.84 | 0.850 | 0.396 |
|  | Female | 19.81±5.86 |  |  |
| Education level | Junior high school or below | 19.71±5.89 | 0.407 | 0.748 |
|  | High school or technical secondary school | 19.82±6.12 |  |  |
|  | Junior college | 19.72±6.70 |  |  |
|  | Bachelor degree or above | 20.56±6.57 |  |  |
| Marital status | Unmarried | 19.95±6.82 | 0.493 | 0.612 |
|  | Married | 20.29±6.18 |  |  |
|  | Other | 17.83±7.31 |  |  |
| Personal monthly income (RMB/Yuan) | <1500 | 19.39±6.14 | 0.177 | 0.912 |
|  | 1500-2999 | 20.37±6.08 |  |  |
|  | 3000-4999 | 20.10±6.61 |  |  |
|  | ≥5000 | 20.24±6.46 |  |  |
| Place of residence | Rural | 19.42±6.50 | -0.930 | 0.353 |
|  | Cities or towns | 20.29±6.39 |  |  |
| Types of medical insurance | Medical insurance | 17.17±7.36 | 0.998 | 0.394 |
|  | Out-of-pocket medical care | 20.31±7.35 |  |  |
| Types of psoriasis | Psoriasisvulgaris | 20.05±6.43 | 0.793 | 0.498 |
|  | Psoriasisarfhropathica | 21.90±4.91 |  |  |
|  | Psoriasispustulosa | 24.67±4.51 |  |  |
|  | Erythrodermicpsoriasis | 19.60±7.44 |  |  |
| Site of skin lesions | Head and face | 21.48±6.64 | 1.633 | 0.182 |
|  | The four limbs | 19.98±6.29 |  |  |
|  | Trunk | 19.30±5.68 |  |  |
|  | The whole body | 19.63±6.39 |  |  |
| Duration of disease (years) | <10 | 22.27±5.69 | 26.692 | <0.001 |
|  | 10-20 | 19.86±6.65 |  |  |
|  | >20 | 16.40±5.62 |  |  |
| Severity of illness | Mild | 24.90±4.22 | 217.837 | <0.001 |
|  | Moderate | 18.81±5.56 |  |  |
|  | Serious | 14.48±3.78 |  |  |
| Whether there is itching | Yes | 19.98±6.40 | -0.753 | 0.452 |
|  | No | 20.62±6.46 |  |  |
| Whether there is a family history | Yes | 21.15±6.14 | 1.450 | 0.148 |
|  | No | 19.87±6.46 |  |  |
